# Biofortification of Plant- and Animal-Based Foods in Limiting the Problem of Microelement Deficiencies—A Narrative Review

**DOI:** 10.3390/nu16101481

**Published:** 2024-05-14

**Authors:** Wojciech Białowąs, Eliza Blicharska, Kamil Drabik

**Affiliations:** 1 Faculty of Medicine, Medical University of Lublin, 20-093 Lublin, Poland; woj.bialowas@gmail.com; 2 Department of Pathobiochemistry and Interdyscyplinary Applications of Ion Chromatography, Faculty of Biomedicine, Medical University of Lublin, 20-093 Lublin, Poland; bayrena@o2.pl; 3 Institute of Biological Basis of Animal Production, University of Life Sciences in Lublin, 20-950 Lublin, Poland

**Keywords:** malnutrition, mineral element deficiency, food enrichment

## Abstract

With a burgeoning global population, meeting the demand for increased food production presents challenges, particularly concerning mineral deficiencies in diets. Micronutrient shortages like iron, iodine, zinc, selenium, and magnesium carry severe health implications, especially in developing nations. Biofortification of plants and plant products emerges as a promising remedy to enhance micronutrient levels in food. Utilizing agronomic biofortification, conventional plant breeding, and genetic engineering yields raw materials with heightened micronutrient contents and improved bioavailability. A similar strategy extends to animal-derived foods by fortifying eggs, meat, and dairy products with micronutrients. Employing “dual” biofortification, utilizing previously enriched plant materials as a micronutrient source for livestock, proves an innovative solution. Amid biofortification research, conducting in vitro and in vivo experiments is essential to assess the bioactivity of micronutrients from enriched materials, emphasizing digestibility, bioavailability, and safety. Mineral deficiencies in human diets present a significant health challenge. Biofortification of plants and animal products emerges as a promising approach to alleviate micronutrient deficiencies, necessitating further research into the utilization of biofortified raw materials in the human diet, with a focus on bioavailability, digestibility, and safety.

## 1. Introduction

The world’s increasing human population is a force propelling food production. However, the significant productivity increases in both animals [1,2] and plants [3] recorded in recent years and achieved through intensive breeding work and the application of intensive fertilization or plant protection products already fails to meet food security requirements for most countries. Predictions of further increases in the demand for food, and thus the incidence of hunger, are not optimistic in this regard, and they indicate that there will be a significant increase in the human population’s requirement for food products by 2050 [4,5].

At the same time, attention is increasingly being drawn to the fact that the term malnutrition refers not only to an insufficient supply of energy or nutrients in the diet but also to vitamin or mineral deficiencies. Increasingly, the phrase “hidden hunger” is being used in this regard, which can affect up to one in three people worldwide [6]. The need to ensure the availability of a mineral supply at an adequate level has not only led to the need to look for methods of enriching food with deficient ingredients but has also influenced changes in the global approach. The Sustainable Development Goals (SDGs) that were developed highlighted the problem of availability not only of food as a whole but also the need for the proper contents of its individual elements, including minerals [7].

Malnutrition is a very complex topic due to the multitude of factors affecting it. Although it may seem that this problem is directly related to hunger, it should be considered in more detail. Indeed, literature data indicate that patients suffering from quality malnutrition also suffer from obesity [8,9]. Studies indicate that obese people suffer from abnormal levels of serum minerals [10], which ultimately disrupt the body’s functioning at various levels. Many works point to the growing problem of obesity in society, including in children. In the context of micronutrients, obesity in children affects the incidence of iron deficiency [11]. Although the problem is not fully understood, an improperly balanced diet or the occurrence of low-grade inflammation caused by overexposure to fatty acids is cited as the cause of these deficiencies [12]. However, iron is not the only microelement deficient in obese patients. Deficiencies of other micronutrients like zinc and copper are also indicated [13]. Although there have been a number of research works on determining the relationship of zinc levels [14,15,16], no univariate relationships have been identified. This makes the problem of micronutrient deficiency more complex because it can also occur in people whose dietary supply of energy and protein components is not only normal but even excessive.

The problem of mineral deficiencies in the human diet is one of the major nutritional problems, along with ensuring a sufficient protein–energy balance, and avitaminosis is listed as one of the most important hunger issues. According to data presented by Prasad [17], deficiencies of just three essential micronutrients (Fe, I, Zn) are responsible for the mortality of nearly half a million children under the age of five annually. These numbers are all the more drastic because mortality caused by micronutrient deficiencies is the result of extreme deficiencies, while even minor micronutrient deficiencies can cause adverse developmental changes in children, leading to burdens of disease [18].

At the same time, it should be noted that, depending on the study, from 17 to more than 20 micronutrients are listed as essential for life and proper maintenance of the body’s homeostasis [17,19,20]. These minerals are supplied in food from both plant and animal sources. However, today, due to a number of factors related to an improperly balanced diet, intestinal absorption disorders or limited bioavailability [21], and reduced nutrient contents in food itself [22], the problem of mineral deficiencies is increasing. Although a number of dietary supplements in various forms are currently available, they are differentiated in bioavailability and cannot be a substitute for a balanced diet [23]. Research demonstrates that the effects of minerals are associated with those of other nutrients, allowing them to provide synergistic effects and achieve improved bioavailability [24].

It is, therefore, necessary to seek solutions that strike a compromise between efficient food production and maintaining a well-balanced food composition. Accordingly, attention has been given to techniques for enriching foods with minerals in order to produce functional foods not only with elevated contents but that also ensure a higher bioavailability of deficient components.

The purpose of this article is to introduce the issue of mineral deficiencies and the use of biofortification as a method for reducing the associated problems. To that end, work related to biofortification of plant materials using different strategies, enrichment of animal raw materials, and limitations of the biofortification method was reviewed.

## 2. The Role of Micronutrients and the Effects of Their Deficiency in the Human Diet

In addition to protein and energy deficiencies, the problem of starvation also involves reduced access to minerals. These are essential for the proper functioning of the body at the molecular, cellular, and systemic levels [25]. Varying physiological processes and nutrition impinge on the adsorption, digestion, and utilization of micronutrients in the body, and the need for minerals itself will also depend on genetics, gender, age, infections, or certain diseases [26]. Although the problem of mineral deficiencies primarily affects developing countries, the situation somewhat affects almost all countries and social groups.

The groups most greatly exposed to malnutrition are pregnant women and children. It is important to note here that the impact of deficiencies both during pregnancy and in the early stages of a child’s life can affect the child’s subsequent normal development [27,28,29]. Admittedly, these shortages can occur in all countries regardless of their level of development, but more cases are reported in countries with lower economic indicators [30].

On the other hand, in the case of developed countries, the problem is that people usually consume high-calorie foods with low nutrient contents and, consequently, low micronutrient densities, which results not only in obesity but also in disease symptoms caused by micronutrient deficiencies [8].

While there have been a number of local studies [31,32,33], there have been few works on global trends. From the data presented by Luo et al. [34], it appears that the global situation is difficult but is characterized by considerable variability. Undoubtedly, two groups remain the most vulnerable to micronutrient malnutrition: pregnant women and children. These findings are consistent with the report proposed by Beal et al. [35]. Furthermore, both studies point out the variation in mineral deficiencies, while identifying iron, selenium, iodine, and zinc deficiencies as the biggest global problems. At the same time, lack of a balanced diet and high exposure to stress also cause significant magnesium deficiencies in humans [36].

In terms of mineral supplementation using biofortified foods, the four micronutrients Fe, Zn, Se, and I play the most important roles. They have essential functions in maintaining the body’s homeostasis by acting as cofactors of enzymatic reactions or components of proteins (such as selenoproteins or hemoglobin) or by providing a redox balance.

Iron plays a crucial role in oxygen transport as a component of hemoglobin. Moreover, it also has an important role in regulating cell proliferation and ensuring the oxidative balance. In the case of disorders of iron levels (both deficiency and excess), dysfunctions involving almost the entire body can occur (Table 1). Classic symptoms of deficiency include anemia, decreased cognitive function, or decreased immunity. On the other hand, an excess of this element can influence the occurrence of pathological changes in the liver and muscular system and the formation of neurodegenerative diseases [37,38].

Zinc is a cofactor of many enzymatic reactions. It is also a stabilizing factor for the cellular and membrane structure. In addition, it actively contributes to the synthesis and degradation of carbohydrates, lipids, proteins, and nucleic acids. Zinc is also an essential component in the processes of transcription, translation, and metabolic differentiation of cells. Mild zinc deficiency is common, especially in resource-limited countries, which can lead to reduced growth rates, reduced immunity, and metabolic disorders [39,40,41].

Selenium, a component of selenoenzymes and selenoproteins, is one of the essential micronutrients. It has an antioxidant function and also regulates energy metabolism. Selenium deficiency can affect a number of physiological functions (Table 1), and the primary effects include hair loss, decreased concentration and cognitive function, or decreased immunity. Importantly, selenium at elevated levels has toxic effects, and at a dose of 300 mg/kg body weight, it causes lethal poisoning [42,43].

**Table 1:** Effects of micronutrient deficiencies on selected systems in the human body.

Element Deficiency	Se	Fe	Zn	I
Cardiovascular system	Keshan’s disease, atherosclerosis, hypertension, and congestive heart failure	Heart failure	As a result of its antioxidant properties, deficiency may be correlated with the development of cardiovascular diseases, including atherosclerosis	Indirectly, iodine deficiency leading to hypothyroidism can cause arrhythmias such as bradycardia and atrioventricular block, impaired systolic function, increased left ventricular (LV) diastolic filling, diastolic dysfunction with impaired cardiac relaxation, and atrial stiffness
Nervous system	Muscular dystrophy (multinodular myopathy, rigid spinal muscular dystrophy and desmin-associated myopathy with Mallory bodies), disorders of mental status	Deficient myelinization of the brain and impaired metabolism of monoamines, resulting in deficits in memory/learning and motor skills, but also in emotional and psychological disorders	Maternal zinc deficiency has been associated with severe fetal congenital malformations of the central nervous system and increased maternal morbidity *Acrodermatitis enteropathica*	Iodine deficiency can lead to hypothyroidism, which negatively affects the development of the fetal renal system. Children of iodine-deficient mothers are at risk of cognitive impairment, with cretinism being one of the most serious symptoms
Gastrointestinal system	Hepatopathies	Constipation and bowel problems	Appetite disorders and diarrhea *Acrodermatitis enteropathica*	Increased risk of atrophic gastritis and gastric cancer
Endocrine system	Autoimmune thyroiditis	Iron deficiency anemia (IDA) impairs the metabolism of tar-its (hypothyroidism)	Reduces the concentration of circulating insulin-like growth factor 1 (IGF-1), contributing to growth retardation and hypogonadism * Acrodermatitis enteropathica*	Hypothyroidism, thyroid goiter
References	[44,45]	[46,47,48,49,50,51]	[52,53]	[54,55,56]

In response to the deficiencies of micronutrients and recognizing a need to provide them, new methods of food enrichment are being investigated. One of these is the use of biofortification techniques of raw plant and animal materials, which can offer a valuable source of micronutrients when their contents of micronutrients are increased, thus increasing these micronutrients’ bioavailability.

## 3. Biofortification of Plants and Plant Products

The term food fortification refers to the general enrichment of food at the various stages of its production—both at the stages of plant and animal growth and development, as well as during processing [57,58]. This includes the addition or elevation of not only minerals but also vitamins [59,60,61,62] and fatty acids, especially from the *n*-3 group [63,64] as well as others.

**Figure 1:**
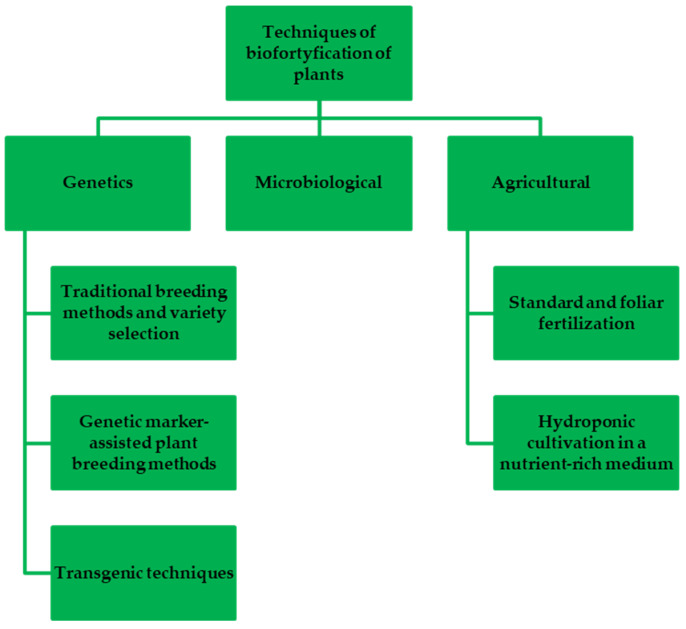
General classification of biofortification methods.

For some foods, especially basic foods such as fruits and vegetables that are consumed in their unprocessed forms, enrichment of the chosen components must occur at the plant growth stage. This is possible and is being exploited using various biofortification techniques (Figure 1). In general, biofortification methods can be divided into agrotechnical biofortification, conventional plant breeding, and breeding methods based on genetic engineering methods [65].

Agronomic biofortification aims to ensure an adequate supply of micronutrients to bring about an appropriate level of their bioaccumulation in plant tissues. Under cultivation conditions, the application of mineral nutrients alone may not yield sufficient results due to the multitude of processes affecting their bioavailability. In general, these are processes related to agrotechnical treatments, including fertilization, as well as those related to the climate or soil conditions. The forms in which micronutrients occur in the soil are also significant. Some of them, such as Fe, Cu, or Mn, are characterized by high mobility and bioavailability to plants in acidic environments, while Se or Mo, for example, is available to plants in alkaline soils [66]. An additional element in this regard is the interactions between micronutrients themselves, which can affect the availability of individual elements on a competitive basis. These aspects result in the use of diverse forms of micronutrients for biofortification under agrotechnical conditions (Table 2).

In recent years, there has been a significant increase in hydroponic cultivation, where a higher level of control over the course of biofortification is possible due to the use of liquid media. Previous research work has achieved effective biofortification in hydroponic cultures of plants such as lettuce [78,79], spinach [80], or wild rocket [81]. The biofortification of microplants has been gaining importance in recent years. In hydroponic crops, enrichment efficacy has been demonstrated for scallion, brasil and cilantro [82], arugula [83], and kale [84]. In hydroponic cultures, two strategies have been adopted to enrich plants through micronutrient cultivation in a rich medium or foliar fertilization, or a combination of the two. The data on the best dosage route are not clear, but it is possible to maximize the effect by achieving synergistic effects especially with the use of dual biofortification [84]. Regardless of the method chosen, the use of hydroponic cultivation techniques, thanks to the control of medium conditions, makes it possible to ensure high bioavailability of the micronutrients used.

Microbial biofortification aims to improve the availability of micronutrients in the soil environment through the action of microorganisms. One case of this type of interaction is the ability of certain bacteria to dissolve zinc compounds, increasing zinc’s availability to plants [85]. This is a method that does not act to increase the content or bioaccumulation capacity of the elements in the plant tissue, but which allows for an improvement in the microelements available in the environment.

Regardless of the additives used in conventional or hydroponic cultivation, a necessary condition for a satisfactory level of biofortification is the ability of plants to sorb and accumulate the desired micronutrients. In this context, it is necessary to carry out breeding work to obtain varieties or lines of biofortified plants. This is possible both through traditional breeding and using genetic engineering methods.

In the case of traditional breeding, the possibility of increasing the mobilization of important micronutrients into wheat and corn grain has been confirmed [86]. Nonetheless, the authors point out that, despite the genetic potential, the final results obtained for micronutrient contents can vary significantly depending on the bioavailability of these nutrients during plant growth and development. Saltzman et al. [87], on the other hand, point out that there is both breeding and nutritional evidence for changes in dietary mineral deficiency levels following the introduction of zinc wheat and millet or iron beans into production. In addition, varieties of other plants characterized by high accumulation of deficient components like rice are also being sought [88]. Simultaneously, there is a trend indicating a higher bioaccumulation capacity of mineral nutrients for more primary varieties. These findings have been confirmed for wheat [89] as well as barley and oats [90]. Thus, work on biofortification based on classical breeding methods can bring a double benefit—reducing nutrient deficiencies and promoting crop biodiversity.

With the development of biotechnology and processing of genetic material, a new area of work on biofortification based on genetically modified (GM) plants has been developed. Although the cultivation of GM crops is already commonplace worldwide [91], it still remains relatively controversial and, in Europe, almost socially unacceptable [92]. However, it should be noted that the solutions introduced hitherto, such as the widely known “Golden Rice”, have contributed to improving the malnutrition situations in their growing regions [93]. Golden rice is one of the most recognizable examples of increasing the level of bioaccumulation of components deficient in plant tissues through genetic modification. Due to the local rice varieties’ inability to accumulate sufficient provitamin A, two provitamin A pathway genes were introduced [94]. The high bioavailability of provitamin A from golden rice was confirmed, significantly reducing vitamin A deficiencies in local populations [95]. Furthermore, through research, in addition to increasing the contents of bioactive components, the ability to mobilize and accumulate minerals has also been improved. Although modification strategies have varied, they mainly focus on bioaccumulation effects and limiting potential toxic effects due to over-concentration. For iron deficiency, the introduction of soybean ferritin genes into rice [96,97] and wheat [98] has proven to be an effective method. Another strategy in this area has been to reduce inhibitors of iron absorption like phytic acid by introducing genes encoding phytase [99]. In the case of micronutrients with potentially toxic effects, it has proven necessary to introduce genes that increase plant tolerance to their exposure. An excellent example in this regard is selenium. Although it is an important micronutrient, it exhibits toxic properties in high doses. Attempts to modify genomes in this case were based on the introduction of genes responsible for increasing tolerance to selenium concentrations and increasing selenate uptake and conversion of selenates to methylselenocysteine [100,101]. The research work has also concerned other minerals and plants, like work on increasing the bioaccumulation capacities for zinc and copper in rice grains [97]. Despite the obvious potential of bioengineering methods in enhancing mineral abundance in deficient raw plant materials, the existing social resistance presents a significant limitation. However, in areas where such social problems do not exist, these methods have the potential to significantly reduce the problems of malnutrition.

## 4. Biofortification of Food of Animal Origin

Foods of animal origin may also be subject to enrichment. In response to the high demand for vitamins A and D, there are commercially available animal products containing these vitamins, such as milk [102] and dairy products [103], as well as eggs [104]. A similar situation applies to the enrichment of animal products with polyunsaturated fatty acids, with the most common method being the addition of vegetable oils (rich in DHA) to processed meat products [105,106] or their use as feedstock. Studies confirm the effectiveness of such treatments for eggs [107,108] and meat [109]. Regarding the practical elements of animal raw material enrichment, it should be mentioned that the use of raw feed materials is common. In addition, aimed at the reduction of vitamin D deficiency, a project entitled “Sunshine Eggs” was carried out. The positive results obtained allowed the raw material to be implemented commercially under the project “Sunshine Eggs 2” [110].

In the context of mineral deficiencies, due to the complexity of the transformations taking place in animal bodies, the enrichment process is somewhat more difficult. The most common interventions with this purpose are in table eggs. These involve the addition of chelated forms of micronutrients to feed [111,112] or organic and inorganic compounds [113]. Recently, there has also been growing interest in the use of nanoparticles, mainly zinc, as an effective method of increasing the content of an element in table eggs [114,115].

Particularly noteworthy, however, are methods based on “dual” biofortification, which use previously enriched plant materials as a source of micronutrients. An excellent example in this regard is the use of microalgae enriched with elements deficient in the diets of laying hens. In their study, Michalak et al. [116] found higher levels of bioaccumulation of copper and manganese compared to the dietary control. A similar effect was obtained when using biofortified soybean seeds at the germination stage [117]. Thus, it seems promising to use not only plant but also raw animal materials as a solution to the problem of nutrient deficiencies in the human diet.

## 5. Bioavailability of Minerals from Biofortified Raw Materials

**Figure 2:**
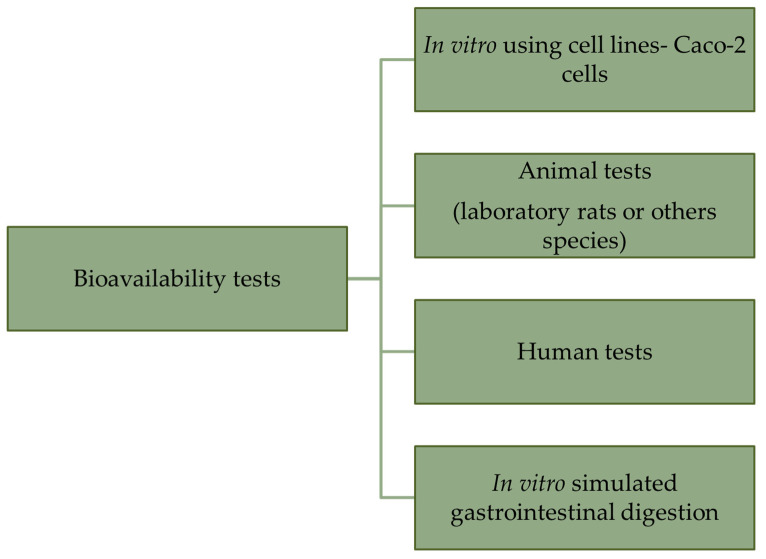
Tests used in bioavailability studies.

The process of biofortification is aimed at providing the highest possible levels of micronutrient concentrations to meet the final demands for these nutrients. For the human diet, the amount of direct research on this topic is relatively limited, especially in the context of human nutrition. To date, evidence cited by Yang et al. [118] indicates a reduction in deficiency-related diseases in the Chinese population following the introduction of crops that accumulate elevated amounts of deficient micronutrients (biofortification through breeding and agronomic practices), but direct data are unfortunately lacking. The performance of bioavailability tests is much less common due to their relatively high cost and the ethical issues associated with conducting tests on humans or using animals. In general, in the available literature, there are four main methods for testing the bioavailability of minerals from biofortified foods (Figure 2).

The most common models are in vitro simulated gastrointestinal digestion and tests using cell lines that are models for studying intestinal epithelial absorption, such as Caco-2 cells. However, these tests have some limitations. In the case of in vitro simulated gastrointestinal digestion, it is possible to analyze the fraction released from the food matrix. Nonetheless, this only indirectly testifies to bioavailability, as there is no way to verify ingredient uptake at the intestinal absorption stage. This is why tests of this type are most often combined with tests using monolayers of Caco-2 cells. On the other hand, Caco-2 cells are tumor cells (colorectal adenocarcinoma) and thus there are limitations to their use in studies of the bioavailability of selected micronutrients. Notably, it was shown that iodine-biofortified lettuce exhibited anti-cancer effects [119], as well as selenium-enriched cauliflower [120]. Thus, there is a reasonable suspicion that there are factors limiting the effectiveness of this method of bioavailability analysis for these trace elements. Nevertheless, both in vitro simulated gastrointestinal digestion and Caco-2 cells are valuable diagnostic tools in bioavailability analysis (Table 3).

Research using animals is far less common, due to its cost-intensive nature. To date, research work has used laboratory animals (rats, usually Wistar) but also other species (Table 3). Depending on the element to be analyzed, the hemoglobin depletion–repletion method (Fe), analysis of micronutrient contents in serum, urine, and feces, and stable isotopes have been used [121]. It is also possible to perform micronutrient content analysis in tissues [137,138], which can allow for indirect determination of the biofortification efficacy in the context of animal viability.

For human studies, stable isotope methods and analysis of hemoglobin levels and plasma micronutrient contents have been used most often [139]. Unfortunately, the results in humans often differ from those determined for model or animal studies, so further research work is needed on the effects of biofortified foods in supplementing micronutrient deficiencies in humans.

## 6. Commercialization and Restrictions on the Introduction of Biofortified Raw Materials

Despite the potential of biofortified raw materials in combating mineral deficiencies in humans, as confirmed by numerous reports mainly on iron [125,140,141], for their complete commercialization to be possible, it must be economically viable. A number of international and local programs like HarvestPlus have been established to support initiatives to market biofortified varieties. The goal of combating mineral deficiencies alone is an insufficient motivator, especially in developing countries, but the introduction of subsidies for biofortified crops can support their commercialization [142]. To date, crops obtained through classical breeding or molecular breeding have been introduced into commercial cultivation. Among them are rice (including varieties enriched in iron and zinc), wheat with increased iron absorption, and others like several varieties of millet, sorghum, and lentils [143].

Although the biofortification process itself raises crop costs, associated with both fertilization and long-term breeding of plants through conventional breeding methods or the need to test plants bred by molecular methods, its profitability should be considered in the long term. For this type of analysis, the DALY (disability-adjusted life years) indicator is most often used. This is suitable because of the risk of serious developmental disorders, especially in the context of an insufficient supply of micronutrients prenatally and during early childhood, as mentioned earlier in this article. As Sheoran [143] reported in their review, for many countries using biofortification strategies, the values of this indicator revealed that the process can be considered highly cost-effective.

However, the impact of biofortification of plants and animal raw materials should also be considered in the context of the environment. Particularly in the context of enrichment methods based on boosting plants with soil and foliar fertilization, there is a risk of disturbance to the soil environment, although foliar fertilization is thought to reduce this problem [144]. Theoretically, the promising results for hydroponic cultivation could allow for an effective biofortification process and cultivation even in areas where soil resources do not allow it. Unfortunately, hydroponic cultivation requires significant amounts of water, the availability of which is already severely limited in some regions of the world, and their prospects point to further problems with its availability [145]. The most applicable approach, therefore, seems to be breeding. In this regard, given the time that traditional breeding takes, it seems necessary to improve awareness and increase public acceptance of plants obtained from molecular breeding.

In the context of biofortification, increasing the concentration levels of micronutrients in plants is only half the battle. This is because it is necessary to supply them in the right amounts to the human body. Processing and digestion processes limit the amount of available micronutrients. For example, work on bean processing showed that despite the high initial mineral content, the concentrations changed after processing [146]. Similar conclusions were also reached by Ciccolini et al. [147], who analyzed the iron and zinc contents of flours extracted from biofortified and untreated wheat varieties. In whole grain and whole wheat flour, significantly higher concentration levels of these components were obtained than in white flour. This indicates the need not only for enrichment of raw materials but also for effective processing technology.

## 7. Biofortification as a Tool and Not a Remedy for the Problem of Hidden Hunger

Despite numerous studies indicating the high potential of biofortification in reducing microelement deficiencies in the human diet, it should be understood that it is a tool and not a remedy. First of all, it should be accepted that, like supplementation in the form of commercially available supplements, biofortified products cannot offer a substitute for a balanced diet. Literature data clearly indicate that a properly varied diet contributes to ensuring an adequate supply of micronutrients [148]. Studies analyzing dietary diversity show a clear trend in this regard. Lack of a varied diet can contribute to micronutrient deficiencies, including in groups that are particularly vulnerable to these deficiencies like pregnant women [149,150]. A more varied diet should, therefore, be adopted as the primary solution to hidden hunger in the context of micronutrients [151,152].

Although the important role of nutrient deficiencies in hidden hunger has led to an increased scope for food biofortification, as van Ginkel and Cherfas [153] point out, biofortification by itself is not enough and suggesting that the consumption of biofortified raw materials will eliminate hidden hunger indicates a significant oversimplification of the problem. The authors additionally raise issues related to the large disparity in funding for research related to biofortification and analyzing alternatives. It should also be noted that the increased interest in biofortified varieties that have been marketed to date may exacerbate the biodiversity problem in the agricultural environment [153].

Moreover, in the context of micronutrients themselves, it should be noted that “every coin has two sides”. Over-supply of these nutrients can significantly contribute to negative health effects [66]. The negative effects of an excessive supply of micronutrients can include a number of disease entities like neurodegenerative changes [154], diabetes [155], and endocrine and reproductive system disorders [156]. It is, therefore, necessary to maintain appropriate levels of these nutrients, without exceeding the standards for the recommended intake. In the case of biofortified foods, the amount of direct research to date on bioavailability is small, and the issue should be considered more closely.

This is all the more important because, as already indicated, the amount of data on the bioavailability of micronutrients from biofortified products is insufficient. At the same time, it should be remembered that some of the micronutrients show competition in terms of absorption, as is the case with iron and manganese [157], iron and zinc [158], or many others. With this in mind, it should be assumed that in order to fully address the problem of hidden hunger, a balance must be maintained between the introduction of biofortified products, a balanced diet, and factors that limit the absorption of deficient micronutrients.

## 8. Conclusions

The issue of mineral deficiencies in the human diet is a significant nutritional problem, manifesting not only in the form of various diseases, developmental changes, or illnesses but also a significant increase in mortality, especially in children. One solution to this problem seems to be offered by biofortified plant and animal raw materials. Although a lot of data on food enrichment are available in the literature, few reports focus on the bioavailability of micronutrients. At the same time, experimental data from in vitro and in vivo studies in animal models indicate significant potential for biofortification methods in the supplementation of deficient components. Biofortification shows high potential in mitigating the global effects of so-called “hidden hunger”, although further research work is needed on the application of biofortified raw materials to the human diet, with emphases on their digestibility, bioavailability, and safety. At the same time, biofortification should be treated as a tool for alleviating hidden hunger and not as a solution, recognizing its impact not only on the human body but also in the changes it brings to the entire food production sector.

## Figures and Tables

**Table 2:** Literature data on bioavailable forms of elements and compounds used for their biofortification.

Element	Forms Available for Plants	Chemicals Used in Biofortification	Source
Fe	Fe^2+^, Fe^3+^, iron chelates	FeSO_4_, nanoparticles, organic combinations (Fe-HBED, Fe-DTPA), iron citrate	[67,68,69]
Zn	Zn^2+^, zinc chelates, zinc in complex form—for example, in combination with amino acids (Zn + glycine)	Zn-EDTA, Zn + amino acids (ZN + AA), ZnSO4·7H2O, ZnO nanoparticles	[70,71]
I	I^−^, IO_3_^−^, organic combinations, CH_3_I	I^−^, IO_3_^−^, iodine–chitosan complex, solutions of iodoquinolines	[72,73,74,75]
Se	SeO_4_^2−^, SeO_3_^2−^, organic forms like selenocysteine, selenomethionine	SeO_4_^2−^, SeO_3_^2−^, synthesized and biosynthesized selenium nanoparticles	[76,77]

**Table 3:** Review of bioavailability testing methods.

Microelement	Biofortified Material	Bioavailability Test	Result	Reference
Fe	Cowpea (*Vigna unguiculata* L. *Walp*)	Wistar rats	No differences in hemoglobin levels, but hemoglobin levels similar to the ferrous-sulfate-supplemented group	[121]
	Red mottled beans (*Phaseolus vulgaris* L.)	Caco-2 cells/animal test on chickens	For Caco-2 cells, significantly higher ferritin levels were found after the use of biofortified beans with high iron concentrations For chickens, there was a significant increase in hemoglobin content for birds fed feed with biofortified iron, with no difference in liver ferritin levels for the groups tested	[122]
	Transgenic and wild-type indica rice	Caco-2 cells	Increased bioavailable iron levels in biofortified rice compared to wild type	[123]
	Carioca bean	Caco-2 cells/Wistar rats/humans	No differences in in vitro tests (ferritin levels); higher bioavailability of iron in biofortified beans than control in a study in rats; no effect on human nutrition	[124]
	Fe-biofortified beans	Humans	The group supplemented with biofortified beans had a significantly greater increase in hemoglobin and serum ferritin compared to the control group	[125]
Zn	Rice (*Oryza sativa* L.)	Caco-2 cells/Sprague–Dawley rats	Increased zinc absorption from biofortified rice in rats	[126]
	Pearl millet (*Cenchrus americanus*)	Humans	Higher zinc absorption from biofortified millet compared to non-biofortified group	[127]
	Canon bean and Pontal bean	Humans	No impact of zinc level in serum	[124]
Se	Lettuce (*Lactuca sativa* L.)	Caco-2 cells	Improved Se assimilation from selenate biofortified lettuce.	[128]
	Microalga (*Chlorella sorokiniana*)	Mice (Mus musculus)/in vitro simulated gastrointestinal digestion	An increase in Se bioavailability in mice was observed, but only at a low dose, and high levels in the kidneys, associated with excretion, in simulated digestion showed high (81%) Se availability, especially in the form of selenomethionine	[129]
	Radish seedlings	In vitro simulated gastrointestinal digestion	High bioavailability (exceeding 85%) in the test material after simulated in vitro digestion	[130]
	Wheat	Caco-2 cells	Despite the high Se content of biofortified wheat, only 19.6% of Se was absorbed by cells	[131]
I	Rice and wheat	In vitro simulated gastrointestinal digestion	High release rate of iodine from the matrix in in vitro digestion	[132]
	Celery and pak choi	In vitro simulated gastrointestinal digestion	The loss of iodine content during processing (soaking, cooking) was determined, and a high bioavailability was found after simulated digestion	[133]
	Biofortified vegetables (potatoes, carrots, cherry tomatoes, and green salad)	Humans	Increase in urinary iodine content as a diagnostic indicator in this study of iodine deficiency disorders	[134]
	Carrots	Wistar rats	Higher iodine levels in urine, feces, and tissues	[135]
	Lettuce	Wistar rats	Higher iodine levels in urine, feces, and tissues	[136]
